# Genetic risk between the *CACNA1I* gene and schizophrenia in Chinese Uygur population

**DOI:** 10.1186/s41065-017-0037-1

**Published:** 2017-07-17

**Authors:** Wei Xu, Yahui Liu, Jianhua Chen, Qingli Guo, Ke Liu, Zujia Wen, Zhaowei Zhou, Zhijian Song, Juan Zhou, Lin He, Qizhong Yi, Yongyong Shi

**Affiliations:** 1 Department of biology, School of Life Science, Anhui Medical University, 81 meishan road, Hefei, Anhui 230031 China; 2 Bio-X Institutes, Key Laboratory for the Genetics of Developmental and Neuropsychiatric Disorders (Ministry of Education) and the Collaborative Innovation Center for Brain Science, Shanghai Jiao Tong University, Shanghai, 200030 People’s Republic of China; 3 Shanghai Key Laboratory of Psychotic Disorders, Shanghai Mental Health Center, Shanghai Jiao Tong University School of Medicine, Shanghai, 200030 People’s Republic of China; 4 Psychological Medicine Center, The First Affiliated Hospital of Xinjiang Medical University, 137 Liyushan South Road, Urumqi, Xinjiang, 830054 China

**Keywords:** Schizophrenia, *CACNA1I* gene, Case-control study, Uighur Chinese

## Abstract

**Background:**

Schizophrenia (SCZ) is a common mental disorder with high heritability, and genetic factors play a major role in the pathogenesis. Recent researches indicated that the *CACNA1I* involved in calcium channels probably affect the potential pathogenesis of SCZ.

**Results:**

In this study, we attempted to investigate whether the *CACNA1I* gene contributes the risk to SCZ in the Uighur Chinese population, and performed a case-control study involving 985 patient samples and 1218 normal controls to analyze nine SNPs within the *CACNA1I* gene. Among these sites, six SNPs were significantly associated with SCZ in the allele distribution: rs132575 (adjusted *P*_*allele*_ = 0.039, OR = 1.159), rs713860 (adjusted *P*_*allele*_ = 0.039, OR = 0.792), rs738168 (adjusted *P*_*allele*_ = 0.039, OR = 0.785), rs136805 (adjusted *P*_*allele*_ = 0.014, OR = 1.212), rs5757760 (adjusted *P*_*allele*_ = 0.042, OR = 0.873) and rs5750871 (adjusted *P*_*allele*_ = 0.039, OR = 0.859). In addition, two SNPs turned to be risk factors for SCZ not only in the allele distribution, but also in the genotype distribution: rs132575 (adjusted *P*_*genotype*_ = 0.037) and rs136805 (adjusted *P*_*genotype*_ = 0.037).

**Conclusions:**

Overall, the present study provided evidence that significant association exists between the *CACNA1I* gene and SCZ in the Uighur Chinese population, subsequent validation of functional analysis and genetic association studies are needed to further extend this study.

## Background

Schizophrenia (SCZ) is one of enigmatic, complex psychotic mental disease that characterized by abnormalities in the perception or expression of reality, causing a substantial burden on patients and public expenditure [1, 2]. The lifetime prevalence of SCZ is generally estimated to be 1%, and genetic risks account for up to 80% occurrences [3]. This chronic disorder poses series of typical manifestations resembling auditory hallucinations, delusions, and behavioral dysfunction [4, 5]. A lot of crucial developments in neuropathology, epidemiology, and medications are emerged, triggering better identification of etiology and effective therapeutics. Analysis of the genetic epidemiologic in family, twin, and adoption, the conclusion suggest that hereditary loci for which linkage to the SCZ play a critical role in the development of the disease [6].

With the deepening research of gene detection and disease mechanism, *CACNA1I* (calcium voltage-gated channel subunit alpha1 I) has been identified as a candidate gene for SCZ. Recently, a primary GWAS conducted by the Psychiatric Genomics Consortium-Schizophrenia Workgroup (PGC-SCZ) has made encouraging progress in identifying genetic susceptibility loci, and the *CACNA1I* gene is reported as a new locus for SCZ in Caucasian [7]. *CACNA1I* is located at 22p13.1, spanning about 118 kb genomic region, and consists of 38 exons. This gene encodes Cav3.3 isoform that contains a pore-forming alpha subunit, and the coding product of *CACNA1I* is a member of low-threshold (T-type) Ca^2+^ channels [8, 9]. The *CACNA1I* gene is abundantly expressed in the thalamic reticular nucleus, and delineates the distinctive physiological properties of neuronal firing [10, 11]. There are three subtypes of low threshold voltage-activated T-type Ca^2+^ channels have been implicated and designated α_1G_ (Cav3.1), α_1H_ (Cav3.2) and α_1I_ (Cav3.3) by previous reports, which endow typical kinetic features and involve in different signatures of T-currents, respectively [12]. In view of the exploration of the thalamic reticular and relay neurons activities, increasing results point to Cav3.1 and Cav3.2 channels represent short burst firing and small conductance, while Cav3.3 leads to slower activation and inactivation [13, 14].

The normal physiological activities of human beings need to be maintained through the action potential discharge of specific ion channels. Ion exchange is responsible for the level of intracellular Ca^2+^, carry out a series of electrical, chemical, and physical function [15]. Evidence demonstrates that *CACNA1I* mRNA is ubiquitously expressed in brain regions, and Cav3.3 channel provoked by small membrane depolarization can elicit spontaneous discharge. The channel encoded by *CACNA1I* plays a central role in the thalamic spindle generator [16], alongside reduced sleep spindles associate with SCZ [17]. Abnormalities of sleep spindles and disturbances in thalamic neurons, are found in people with schizophrenia. It is noteworthy that the encode proteins has been reported can meet the druggable target of SCZ [18]. Moreover, T-type calcium channels have been shown to be a crucial cause of insomnia and neuropathic pain [19]. There is evidence that a single copy of Chr22:39665939G > A *CACNA1I* triggers calcium channel disorder and is associated with the pathogenesis of SCZ [20]. These profound findings have prompted us to open up promising research idea that *CACNA1I* might regulates signaling pathways in SCZ.

Uygur is one of the minority nationalities in China, and mainly distributes in Xinjiang Province. The region located in the northwest border area of China, and the hinterland of the Eurasian continent. As a part of the ancient Silk Road, the mutual migration between the countries, the typical diets, and the different lifestyles play the important role in shaping the genetic structure [21, 22]. The Uygur populations therefore are results of admixture of Han Chinese and Western Europe [23], and also is the highlight of the current study.

To date, there have been no studies that *CACNA1I* SNPs association with SCZ in the Uygur Chinese population reported, so it is the first study which performed *CACNA1I* in the Uygur Chinese population. A total of nine SNPs were selected in *CACNA1I*, including eight tag SNPs which were examined to provide a good coverage of this region, and one positive SNP which identified from a genome-wide association study was selected [24].

## Methods

### Subjects

In total, 985 unrelated patients with SCZ (612 males and 373 females), and 1218 control individuals (629 males and 589 females) were enrolled from Xinjiang Province. The mean age of SCZ cases was 39.45 years (±12.12), and normal controls was 43.07 years (±13.14). The data was illustrated as Table 1.

**Table 1:** Demographic detail of sample set

	Patients with schizophrenia		Healthy controls	
Total sample(N)	985		1218	
	Male	Female	Male	Female
	612	373	629	589
Mean age ± SD	39.45 ± 12.12		43.07 ± 13.14	

All eligible subjects selected were the native population of Xinjiang province. Clinical diagnosis were carried out in strict accordance with DSM-IV criteria (Diagnostic and Statistical Manual of Mental Disorders, the fourth edition) based on SCID-I (Structured Clinical Interview for DSM-IV Axis I Disorders) by interviewed with two independent psychiatrists. The healthy controls were randomly selected from the general Uighur population. All participants signed informed consent. This study obtained the consent of the local ethnic ethics, and undertaken the support of its support.

### Genomic assay

**Fig. 1:**
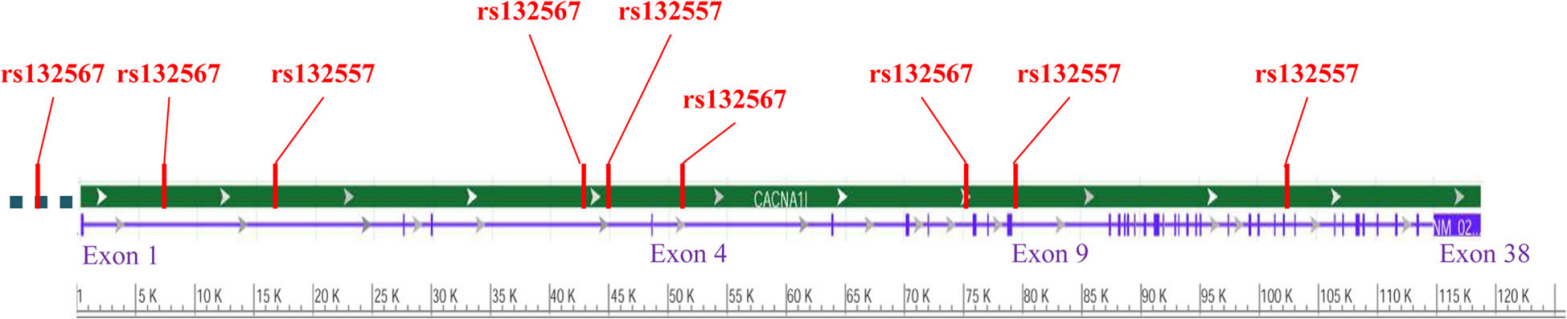
Relative positions in gene *CACNA1I* of nine SNPs

According to QuickGene DNA whole blood kit L (FUJIFILM), genomic DNA was isolated from the peripheral blood of the subjects. Eight tag SNPs (rs132567, rs738168, rs713860, rs11705208, rs132575, rs136805, rs5757760, rs5750871) are obtained through Haploview software version 4.2, with pair-wise r^**2**^ threshold ≥0**.**5 and minor allele frequency ≥ 0.05 [25]. Besides, we put a positive site of the previous research (rs9607658) into the experiment. The specific information of these 9 SNPs is listed in Table 2, while, the nine SNPs in the relative position of *CACNA1I* gene is also shown in Fig. 1. All samples were subjected to genotyping by the Sequenom MassARRAY matrix-assisted laser desorption ionization-time of flight (MALDI-TOF) mass spectrometry platform (Sequenom Inc., San Diego, CA).

**Table 2:** The information of 9 SNPs in *CACNA1I* gene

SNP ID	rs9607658	rs132567	rs132575	rs713860	rs738168	rs136805	rs11705208	rs5757760	rs5750871
Position	39,561,735	39,577,521	39,586,716	39,612,821	39,615,692	39,622,207	39,646,048	39,648,397	39,673,444
Function	intron	intron	intron	intron	intron	intron	intron	intron	intron
Polymorphism	C/T	A/G	C/T	C/T	A/G	C/T	C/T	C/T	A/G

### Statistical analysis

Powerful SHEsis software provides a set of processing parameters for maximum benefit, including allele and genotype frequencies, Hardy-Weinberg equilibrium, association tests and haplotype analysis (http://shesisplus.bio-x.cn/SHEsis.html) [26, 27]. This is a comprehensive platform for processing association study, and perform expectation maximization algorithm in haplotype reconstruction and frequency estimation. Allele and genotype frequencies refer to the percentage of allele and genotype in a population, and show the diversity and abundance of the gene in a population. FDR correction is a conservative method to explain multiple comparisons. All outputted tests were two-tailed, the *P* value standard of the statistical significance were set to be less than 0.05.

## Results

### Single site analysis

The genotype *P* values of the 9 SNPs in Hard–Weinberg equilibrium test (HWE) were all larger than 0.05 in both patients and healthy controls. So they all did not deviate from Hard–Weinberg equilibrium, and demonstrated the genetic properties of this sample population remained relatively stable. Call rates of all loci exceeded 99% in all samples. Detailed information is referenced in Table 3.

**Table 3:** The call rate (%) and HWE test of 9 SNPs in SCZ patients and control

SNP ID	rs9607658		rs132567		rs132575		rs713860		rs738168		rs136805		rs11705208		rs5757760		rs5750871	
	case	control	case	control	case	control	case	control	case	control	case	control	case	control	case	control	case	control
call rate%	0.996		0.997		0.996		0.996		0.997		0.991		0.997		0.992		0.992	
HWE-P	0.904	0.654	0.549	0.222	0.231	0.483	0.515	0.929	0.729	0.955	0.973	0.837	0.658	0.999	0.999	0.989	0.58	0.78

In Table 4, all the allele and genotype *P* values for the 9 SNPs in the patient samples and normal controls are shown. rs132575 and rs136805 were significant in both allele and genotype distributions [rs132575: adjusted *P*_*allele*_ = 0.039, adjusted *P*_*genotype*_ = 0.037; rs136805: adjusted *P*_*allele*_ = 0.014, adjusted *P*_*genotype*_ = 0.037]. In addition, rs713860, rs738168, rs5757760 and rs5750871 were significantly associated with SCZ in the allele distributions [rs713860: adjusted *P*_*allele*_ = 0.039, OR[95% CI] = 0.792[0.652–0.963]; rs738168: adjusted *P*_*allele*_ = 0.039, OR[95% CI] = 0.785[0.651–0.947]; rs5757760: adjusted *P*_*allele*_ = 0.042, OR[95% CI] = 0.873 [0.773–0.985]; rs5750871: adjusted *P*_*allele*_ = 0.039, OR[95% CI] = 0.859 [0.76–0.97]]. It is notable that rs738168 showed genotypic significance with SCZ before FDR correction [*P*_*genotype*_ = 0.03, *P*_*genotype*_ = 0.084 after FDR correction].

**Table 4:** Allele and genotype frequencies of 9 Loci in SCZ

SNP ID	Alleles		OR [95% CI]	*P*-value	P-FDR	Genotypes			*P*-value	P-FDR
rs9607658	T(freq)	C(freq)				T/T(freq)	T/C(freq)	C/C(freq)		
Case	618(0.314)	1350(0.685)	1.101 [0.968 ~ 1.253]	0.142	0.178	94(0.095)	430(0.436)	460(0.467)	0.218	0.245
Control	711(0.293)	1711(0.706)				111(0.091)	489(0.403)	611(0.504)		
rs132567	A(freq)	G(freq)				A/A(freq)	A/G(freq)	G/G(freq)		
Case	1073(0.545)	895(0.454)	0.917 [0.814 ~ 1.034]	0.159	0.178	284(0.288)	505(0.513)	195(0.198)	0.054	0.084
Control	1271(0.523)	1155(0.476)				348(0.286)	575(0.474)	290(0.239)		
rs132575	A(freq)	G(freq)				A/A(freq)	A/G(freq)	G/G(freq)		
Case	1295(0.658)	673(0.341)	1.159 [1.021 ~ 1.316]	*0.021*	*0.039*	414(0.42)	467(0.474)	103(0.104)	*0.008*	*0.037*
Control	1674(0.69)	750(0.309)				587(0.484)	500(0.412)	125(0.103)		
rs713860	C(freq)	T(freq)				C/C(freq)	C/T(freq)	T/T(freq)		
Case	1779(0.904)	187(0.095)	0.792 [0.652 ~ 0.963]	*0.019*	*0.039*	808(0.821)	163(0.165)	12(0.012)	0.056	0.084
Control	2142(0.882)	284(0.117)				947(0.78)	248(0.204)	18(0.014)		
rs738168	C(freq)	A(freq)				C/C(freq)	C/A(freq)	A/A(freq)		
Case	1763(0.895)	205(0.104)	0.785 [0.651 ~ 0.947]	*0.011*	*0.039*	792(0.804)	179(0.181)	13(0.013)	*0.03*	0.084
Control	2115(0.871)	313(0.128)				920(0.757)	275(0.226)	19(0.015)		
rs136805	C(freq)	T(freq)				C/C(freq)	C/T(freq)	T/T(freq)		
Case	1001(0.509)	965(0.49)	1.212 [1.075 ~ 1.365]	*0.001*	*0.014*	253(0.257)	495(0.503)	235(0.239)	*0.006*	*0.037*
Control	1339(0.556)	1065(0.443)				378(0.314)	583(0.485)	241(0.2)		
rs11705208	C(freq)	T(freq)				C/C(freq)	C/T(freq)	T/T(freq)		
Case	1775(0.901)	193(0.098)	1.039 [0.849 ~ 1.27]	0.708	0.708	803(0.816)	169(0.171)	12(0.012)	0.771	0.771
Control	2198(0.905)	230(0.094)				995(0.819)	208(0.171)	11(0.009)		
rs5757760	T(freq)	C(freq)				T/T(freq)	T/C(freq)	C/C(freq)		
case	766(0.389)	1200(0.61)	0.873 [0.773 ~ 0.985]	*0.028*	*0.042*	149(0.151)	468(0.476)	366(0.372)	0.09	0.116
control	1017(0.422)	1391(0.577)				216(0.179)	585(0.485)	403(0.334)		
rs5750871	G(freq)	A(freq)				G/G(freq)	G/A(freq)	A/A(freq)		
case	748(0.38)	1218(0.619)	0.859 [0.76 ~ 0.97]	*0.014*	*0.039*	150(0.152)	448(0.455)	385(0.391)	0.051	0.084
control	1003(0.416)	1403(0.583)				215(0.178)	573(0.476)	415(0.344)		

Italics represent *P*-values < 0.05

According to the gender of the subjects, the two sample sets were obtained separately. Detailed analysis results are illustrated in Tables 5 and 6. For male samples, there are seven ninths of the genes significantly associated with SCZ. rs132575, rs136805, rs5757760 and rs5750871 showed association towards SCZ in both allele and genotype distributions, meanwhile, rs9607658, rs713860, rs738168 revealed stronger positive results in the allele distributions. Interestingly, there was no significant association between *CACNA1I* and SCZ in the female sample, all the *P* values of 9 SNPs were greater than 0.05.

**Table 5:** SNP analysis in men

SNP ID	Alleles		OR [95% CI]	*P*-value	P-FDR	Genotypes			*P*-value	P-FDR
rs9607658	T(freq)	C(freq)				T/T(freq)	T/C(freq)	C/C(freq)		
Case	394(0.322)	828(0.677)	1.218 [1.025 ~ 1.447]	*0.024*	*0.031*	59(0.096)	276(0.451)	276(0.451)	0.065	0.084
Control	350(0.28)	896(0.719)				49(0.078)	252(0.404)	322(0.516)		
rs132567	A(freq)	G(freq)				A/A(freq)	A/G(freq)	G/G(freq)		
Case	674(0.551)	548(0.448)	0.892 [0.761 ~ 1.044]	0.156	0.176	176(0.288)	322(0.527)	113(0.184)	0.167	0.188
Control	655(0.523)	597(0.476)				172(0.274)	311(0.496)	143(0.228)		
rs132575	A(freq)	G(freq)				A/A(freq)	A/G(freq)	G/G(freq)		
Case	790(0.646)	432(0.353)	1.266 [1.07 ~ 1.498]	*0.005*	*0.013*	247(0.404)	296(0.484)	68(0.111)	*0.008*	*0.02*
Control	873(0.698)	377(0.301)				307(0.491)	259(0.414)	59(0.094)		
rs713860	C(freq)	T(freq)				C/C(freq)	C/T(freq)	T/T(freq)		
Case	1111(0.91)	109(0.089)	0.731 [0.563 ~ 0.95]	*0.018*	*0.028*	507(0.831)	97(0.159)	6(0.009)	0.053	*0.079*
Control	1104(0.881)	148(0.118)				486(0.776)	132(0.21)	8(0.012)		
rs738168	C(freq)	A(freq)				C/C(freq)	C/A(freq)	A/A(freq)		
Case	1101(0.9)	121(0.099)	0.724 [0.564 ~ 0.928]	*0.01*	*0.019*	497(0.813)	107(0.175)	7(0.011)	*0.028*	0.051
Control	1087(0.868)	165(0.131)				470(0.75)	147(0.234)	9(0.014)		
rs136805	C(freq)	T(freq)				C/C(freq)	C/T(freq)	T/T(freq)		
Case	600(0.49)	622(0.509)	1.292 [1.102 ~ 1.513]	*0.001*	*0.013*	149(0.243)	302(0.494)	160(0.261)	*0.007*	*0.02*
Control	688(0.554)	552(0.445)				194(0.312)	300(0.483)	126(0.203)		
rs11705208	C(freq)	T(freq)				C/C(freq)	C/T(freq)	T/T(freq)		
Case	1099(0.899)	123(0.1)	1 [0.769 ~ 1.299]	0.998	0.998	498(0.815)	103(0.168)	10(0.016)	0.315	0.315
Control	1126(0.899)	126(0.1)				505(0.806)	116(0.185)	5(0.007)		
rs5757760	T(freq)	C(freq)				T/T(freq)	T/C(freq)	C/C(freq)		
case	464(0.379)	758(0.62)	0.79 [0.673 ~ 0.929]	*0.004*	*0.013*	86(0.14)	292(0.477)	233(0.381)	*0.004*	*0.02*
control	541(0.436)	699(0.563)				103(0.166)	335(0.54)	182(0.293)		
rs5750871	G(freq)	A(freq)				G/G(freq)	G/A(freq)	A/A(freq)		
case	454(0.371)	768(0.628)	0.794 [0.675 ~ 0.933]	*0.005*	*0.013*	92(0.15)	270(0.441)	249(0.407)	*0.009*	*0.02*
control	530(0.426)	712(0.573)				110(0.177)	310(0.499)	201(0.323)		

Italics represent *P*-values < 0.05

**Table 6:** SNP analysis in women

SNP ID	Alleles		OR [95% CI]	*P*-value	P-FDR	Genotypes			*P*-value	P-FDR
rs9607658	T(freq)	C(freq)				T/T(freq)	T/C(freq)	C/C(freq)		
Case	224(0.3)	522(0.699)	0.968 [0.793 ~ 1.182]	0.755	0.907	35(0.093)	154(0.412)	184(0.493)	0.835	0.835
Control	361(0.306)	815(0.693)				62(0.105)	237(0.403)	289(0.491)		
rs132567	A(freq)	G(freq)				A/A(freq)	A/G(freq)	G/G(freq)		
Case	399(0.534)	347(0.465)	0.96 [0.798 ~ 1.153]	0.664	0.907	108(0.289)	183(0.49)	82(0.219)	0.407	0.808
Control	616(0.524)	558(0.475)				176(0.299)	264(0.449)	147(0.25)		
rs132575	A(freq)	G(freq)				A/A(freq)	A/G(freq)	G/G(freq)		
Case	505(0.676)	241(0.323)	1.024 [0.841 ~ 1.247]	0.806	0.907	167(0.447)	171(0.458)	35(0.093)	0.302	0.808
Control	801(0.682)	373(0.317)				280(0.477)	241(0.41)	66(0.112)		
rs713860	T(freq)	C(freq)				T/T(freq)	T/C(freq)	C/C(freq)		
Case	78(0.104)	668(0.895)	0.891 [0.663 ~ 1.196]	0.443	0.907	6(0.016)	66(0.176)	301(0.806)	0.718	0808
Control	136(0.115)	1038(0.884)				10(0.017)	116(0.197)	461(0.785)		
rs738168	A(freq)	C(freq)				A/A(freq)	A/C(freq)	C/C(freq)		
Case	84(0.112)	662(0.887)	0.881 [0.662 ~ 1.172]	0.384	0.907	497(0.813)	72(0.193)	295(0.79)	0.646	0808
Control	148(0.125)	1028(0.874)				470(0.75)	128(0.217)	450(0.765)		
rs136805	T(freq)	C(freq)				T/T(freq)	T/C(freq)	C/C(freq)		
Case	343(0.461)	401(0.538)	1.085 [0.902 ~ 1.305]	0.384	0.907	75(0.201)	193(0.518)	104(0.279)	0.47	0808
Control	513(0.44)	651(0.559)				115(0.197)	283(0.486)	184(0.316)		
rs11705208	T(freq)	C(freq)				T/T(freq)	T/C(freq)	C/C(freq)		
Case	70(0.093)	676(0.906)	1.067 [0.776 ~ 1.466]	0.687	0.907	2(0.005)	66(0.176)	305(0.817)	0.523	0808
Control	104(0.088)	1072(0.911)				6(0.01)	92(0.156)	490(0.833)		
rs5757760	C(freq)	T(freq)				C/C(freq)	C/T(freq)	T/T(freq)		
case	442(0.594)	302(0.405)	0.993 [0.823 ~ 1.197]	0.943	0.943	133(0.357)	176(0.473)	63(0.169)	0.363	0808
control	692(0.592)	476(0.407)				221(0.378)	250(0.428)	113(0.193)		
rs5750871	A(freq)	G(freq)				A/A(freq)	A/G(freq)	G/G(freq)		
case	450(0.604)	294(0.395)	0.954 [0.79 ~ 1.151]	0.626	0.907	136(0.365)	178(0.478)	58(0.155)	0.563	0808
control	691(0.593)	473(0.406)				214(0.367)	263(0.451)	105(0.18)		

### Linkage disequilibrium

**Fig. 2:**
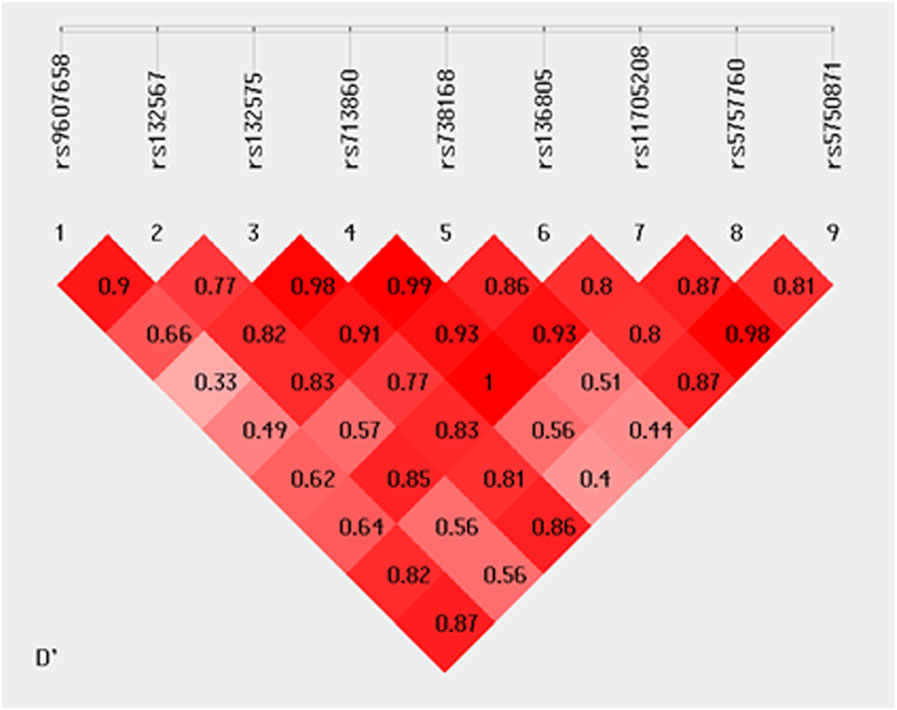
Linkage disequilibrium among 5 SNPs of the *CACNA1I* gene

The pairwise linkage disequilibrium (LD) values among the all investigated SNPs were subjected to calculate in all subjects. A total of 4 haplotype blocks of *CACNA1I* (rs132575-rs713860, rs713860-rs738168, rs713860-rs11705208, rs11705208-rs5750871) were identified when SNPs with D′ > 0.95 were classified in the same block, as presented in Fig. 2.

### Haplotype analysis

There were two haplotypes (A-T: adjusted *P* = 0.038, OR [95% CI] = 0.804 [0.661–0.977]; G-C: adjusted *P* = 0.025, OR[95% CI] = 1.175 [1.035–1.334]) in the block rs132575-rs713860, which were significantly associated with SCZ, haplotype A-T proved to be a protective factor, and haplotype G-C showed it was risk factor. In the block rs713860-rs738168, haplotype C-C and T-A demonstrated protective factor and risk factor of SCZ, respectively (C-C: adjusted *P* = 0.007, OR [95% CI] =1.299 [1.079–1.564]; T-A: adjusted *P* = 0.023, OR[95% CI] = 0.797 [0.656–0.969]). In the block rs11705208-rs5750871, one haplotype C-G presented protective factor of SCZ (adjusted *P* = 0.038, OR [95% CI] =0.873 [0.773–0.986]), another haplotype, C-A, was risk factor after data analysis (adjusted *P* = 0.015, OR [95% CI] = 1.174 [1.042–1.322]). The result of haplotype analysis is suggested in Table 7.

**Table 7:** Haplotype Analysis for *CACNA1I* Gene in SCZ

Blocks with D’ > 0.95	Haplotype	Case(freq)	Control(freq)	Chi ^2^	OR [95% CI]	*P*-value	P-FDR
rs132575-rs713860	A-C	1107(0.563)	1393(0.575)	0.435	0.96 [0.851 ~ 1.082]	0.52	*0.509*
	A-T	187(0.095)	281(0.116)	4.788	0.804 [0.661 ~ 0.977]	*0.03*	*0.038*
	G-C	672(0.341)	745(0.307)	6.216	1.175 [1.035 ~ 1.334]	*0.013*	*0.025*
rs713860-rs738168	C-C	1762(0.896)	2112(0.87)	7.714	1.299 [1.079 ~ 1.564]	*0.006*	*0.007*
	T-A	187(0.095)	283(0.116)	5.161	0.797 [0.656 ~ 0.969]	*0.023*	*0.023*
rs713860-rs11705208	C-C	1588(0.807)	1913(0.788)	2.883	1.136 [0.98 ~ 1.317]	0.091	*0.134*
	C-T	191(0.097)	229(0.094)	0.109	1.034 [0.845 ~ 1.266]	0.757	*0.757*
	T-C	187(0.095)	284(0.117)	5.352	0.794 [0.653 ~ 0.965]	*0.021*	0.062
rs11705208-rs5750871	C-G	745(0.379)	1000(0.417)	4.761	0.873 [0.773 ~ 0.986]	*0.03*	*0.038*
	T-A	191(0.097)	225(0.093)	0.268	1.055 [0.861 ~ 1.292]	0.604	*0.604*
	C-A	1027(0.522)	1172(0.488)	7.041	1.174 [1.042 ~ 1.322]	*0.008*	*0.015*

Italics represent *P*-values < 0.05

## Discussion

SCZ is a genetically complex neuropsychiatric disorder, but the specific etiology of this disease is still vague. SCZ is highly heritable, and the genes that contribute to the disorder play an important role [28]. In this context, we have attempted to confirm an association of *CACNA1I* variants with SCZ. We discovered nine variation sites within the *CACNA1I* locus, as well as one previously studied by Aiden Corvin et al. [24]. This is first study which replicated genetic susceptibility of *CACNA1I* gene in the Uygur Chinese population.

We found nominally association between several SNPs of *CACNA1I* and SCZ. There are four SNPs, rs713860, rs738168, rs5757760 and rs5750871 identified to be associated with SCZ in the allele distributions. In addition, both rs132575 and rs136805 were found to be significantly associated in allelic and genotype analysis. Before FDR correction, rs738168 was associated with schizophrenia in the genotype distribution. Most of the investigated SNPs were positive in our subjects.

rs9607658 was reported as a risk factor for SCZ in population of Ireland in a genome–wide association study (GWAS) by Aiden Corvin et al. (combined *P* = 3.3 × 10^−5^, OR[95% CI] = 1.21[1.10–1.33]) [24]. However, rs9607658 did not confer susceptibility in the present study (adjusted *P* = 0.142, OR[95% CI] = 1.101[0.968–1.253]). This is likely to be caused by racial differences between Uygur and Ireland populations, and the existence of genetic heterogeneity can lead to such a result. A study on Uygur genetic characteristics suggest Uygur population from northern and southern Xinjiang Province share different proportions of ancestors from the European and Han population, so they are the results of admixture the anthropological features of the East and West [29, 30]. The minor allele frequency (MAF = T) in the Han Chinese population is 0.03, whereas in the Ireland population it is 0.54. The results of these two different populations are profound discrepancy, and Uighur population as mixture of the European and Han population also produce certain differences in MAF. Besides, the accuracy of the result is related to the sample size, and the small sample size in this study is used as a limitation for the significant analysis.

In addition, the result has been adopted segregation analysis of sex as a research strategy. We found that male had more susceptibility loci for SCZ, but all the SNPs were negative in the female group. This may be due to a difference in the prevalence and symptoms of psychiatric disorders from a gender standpoint. Previous literature also shows that the existence of significant gender differences in animal models of mental illness [31]. Compared with women SCZ patients, men with SCZ have a high rate of mortality (death, suicide) and earlier onset in the study of gender differences by Mao-Sheng Ran et al. [32]. For the present study, a total of 373 women in the patient sample, 589 women were recruited in the control group. Sample size is a critical factor in gender analysis, thus, there is a need for a larger sample to validate the association between gender and SCZ.

Although these nine SNPs are located in the intron region of *CACNA1I* gene, and they are not directly involved in the biological functions and characteristics of T-type calcium channel, intronic variations may provide some auxiliary cis-acting elements for gene expression regulation, which plays a role in modifying gene transcription efficiency. The protein encoded by *CACNA1I* is widely expressed in the nucleus reticularis thalami, different splice variants can affect the normal discharge of neurons [33]. Besides, we evaluated the protein interaction of *CACNA1I* gene by the version 10.0 of STRING [34], the result showed the *CACNB2* gene involved in SCZ interacts with *CACNA1I* gene, Whether different splice variants or protein-protein interactions, they may confers risk for SCZ.

The *CACNA1I* gene encodes the alpha-1 subunit of the T-type voltage-gated calcium channel Cav3.3, presenting series of function of calcium ion channel that are involved in the neural development and synapse formation [35]. Gene related to Ca^2+^ signaling, such as *CACNA1I* that encode VGCC subunits is associated with schizophrenia and other psychiatric disorders [36]. Evidence suggested that this gene is significantly associated with psychiatric disorders such as autism spectrum disorders. rs5750860, located in *CACNA1I*, has been reported to be associated with autism spectrum disorders by using existing genome-wide association study (GWAS) data and imputation methods [37] . Previous study indicated *CACNA1I* plays a crucial role in spindle activity by participating in the synchronous oscillation of thalamic cortical neurons, and expected to serve as a novel treatment biomarker associated with impaired cognition for individuals with SCZ by treating spindle deficits [17]. The release of neurotransmitters involved in the pathological process of SCZ, and simultaneously there is the research indicated that the *CACNA1I* gene triggers synaptic plasticity in reticular thalamic neurons. Presynaptic neurotransmitter release and postsynaptic receptor signal transduction play an important role in the transmission of information in the brain [38].

## Conclusion

For this study, our efforts on mental illness represent a promising beginning. This is the first time that genetic factors of the *CACNA1I* gene have been verified to be associated with SCZ in the Uygur Chinese population. Obviously, *CACNA1I* plays a key role in the pathogenesis of SCZ. However, the present study remains a major bottleneck in the validation of larger samples, and a larger sample size could be better demonstrate the role of the *CACNA1I* gene in the etiology of schizophrenia. In addition, the Uighur Chinese population has been verified in the present study, and genetic association of other ethnic groups are suggested. Further functional studies of the *CACNA1I* gene are encouraged to conduct, especially in other ethnic groups. All the analysis will facilitate new therapeutic route for SCZ and may provide new insight into the pathogenesis of psychiatric illnesses.

## Abbreviations

*CACNA1I*: Calcium voltage-gated channel subunit alpha1 I
DSM-IV: Diagnostic and Statistical Manual of Mental Disorders, the fourth edition
GWAS: Genome wide association study
HWE: Hardy–Weinberg equilibrium test
LD: Linkage disequilibrium; OR, odds ratio
MALDI-TOF: Matrix-assisted laser desorption ionization-time of flight
PGC-SCZ: Psychiatric Genomics Consortium -Schizophrenia Workgroup
SCID-I: Structured Clinical Interview for DSM-IV Axis I Disorders
SCZ: Schizophrenia
